# Supplementary material for: Prediction of perinatal depression among women in Pakistan using Hybrid RNN-LSTM model
Source: PeerJ Comput Sci. 2025 Feb 26;11:e2673. doi: 10.7717/peerj-cs.2673 (PMC11888857; doi:10.7717/peerj-cs.2673)
Supplement: Supplemental Information 2 [file peerj-cs-11-2673-s002.pdf]

SOCIO-DEMOGRAPHIC QUESTIONNAIRE

DIRECTIONS: Please answer each question as accurately as possible by tick the correct answer or filling in the space provided.

1. Age: _____
2. Gestational Age: _____
3. No. of Sons: ________
4. No. of Daughters: ________
5. Gravida: ________
6. Participant Education: __________
7. Husband Education: __________
8. Working Status: Working Lady/ Housewife
9. Previous Miscarriage: Yes/No
10. Physical Health: Healthy / Disability
11. Sufficient money for basic needs: Yes/No
12. Acceptance of the current appearance: Yes/ No
13. Family System: Nuclear/ Joint
14. Child Male Gender Preference: Yes/ No
15. Relationship with Mother-in-law: Good/ Moderate/Poor
